# Clinical reports of surgical dislocation of the hip with sequestrum clearance and impacting bone graft for grade III_A_-III_B_ aseptic necrosis of femoral head (ANFH) patients

**DOI:** 10.18632/oncotarget.15095

**Published:** 2017-02-04

**Authors:** Chen Yao, Nan Yi, Jirong Shen, Bin Du, Guangquan Sun, Hao Shu, Chao Zhang

**Affiliations:** ^1^ Orthopedics Joint Group, The Affiliated Hospital of Nanjing University of TCM, Jiangsu Province Hospital of TCM, Nanjing, China; ^2^ Department of Orthopaedics, The Second Affiliated Hospital of Soochow University, Soochow University, Suzhou, China

**Keywords:** surgical dislocation of the hip, aseptic necrosis of femoral head, impacting bone graft

## Abstract

**Objective:**

To analyze the treatment effect of surgical dislocation of the hip with sequestrum clearance and impacting bone graft in grade IIIA-IIIB aseptic necrosis of femoral head (ANFH) patients.

**Methods:**

From June 2012 to December 2014, 6 patients (total 8 hips) with grade IIIA-IIIB ANFH were retrospectively followed. All the patients were operated with surgical dislocation of the hip with sequestrum clearance and impacting bone graft. Collapse of the femoral head, healing of the osteotomy, along with Harris hip scores were observed after surgeries.

**Results:**

All the patients were followed up, the osteotomies were all healed. Only 1 of the 8 hips (12.5%) was collapsed by the last follow-up. Harris hip scores were improved from 54.52 ± 8.16 to 80.53 ± 7.62 (P <0.001). The excellent rate was 87.5%.

**Conclusion:**

For grade IIIA-IIIB ANFH patients, surgical dislocation with sequestrum clearance and impacting bone graft could possibly achieve satisfactory clinical benefit, particularly for the young patients.

## INTRODUCTION

Aseptic necrosis of femoral head (ANFH) is one common progressive bone disease [1, 2]. ANFH presents with cartilage surface collapse and could develop into osteoarthritis [1, 2]. It is often caused by abnormal blood supply in femoral head, and mainly seen in patients of 20-50 year-old [1, 2]. The pathological cause of ANFH is still not fully understood, but certainly is multifactorial [3–5]. It is well known that treatment of ANFH is disease stage-dependent. For early-stage (Stage-I/II) patients, femoral head preservation procedures are preferred [3–5]. These procedures include core decompression, muscle pedicle grafting and de-rotational osteotomy, among others [3–5]. Currently, it is technically extremely difficult for hip-preservation treatment of patients with grade IIIA-IIIB ANFH [1, 2]. Yet, that hip-preservation procedure shall represent a substantial achievement in the treatment of this type of disease [1, 2].

Our department, or Orthopedics joint group of the Affiliated Hospital of Nanjing University of Traditional Chinese Medicine (Nanjing, China), has been dedicated to operating hip-preservation surgery for ANFH patients. Clinical applications include that the minimally invasive sequestrum clearance and impacting graft with peroneal sustaining, as well as local administration of traditional Chinese medicine Bushen Huoxue (BSHX) decoction. These clinical procedures have achieved satisfactory clinical benefits for many ANFH patients.

For the young patients with grade IIIA-IIIB ANFH who urgently want hip-conservation procedure, minimally invasive hip-preserving surgery often results in poor clinical outcomes [3–6]. This could possibly due to several limitations of the surgery, including restricted view, un-thoroughly dead bone clearance and many others. It is shown that femoral head collapse rate closes to 50% in two years after surgery [7]. In 2001, Ganz et al.,[8] introduced a procedure of exposure and anterior dislocation of the hip, which theoretically supported hip-conservation treatment of those with high-grade ANFH patients. From Jun. 2012 to Dec. 2014, our department has operated surgeries for 6 grade IIIA-IIIB ANFH patients (total 8 hips), using the surgical procedure of dislocation of the hip with sequestrum clearance and impacting bone graft. With max protection of blood perfusion of femoral head, sequestrum clearing and graft impacting in direct sight have achieved satisfied clinical outcomes in a short time. Now clinical data were retrospectively analyzed, and were reported in the current study.

## MATERIALS AND METHODS

### Ethics

Clinical procedures and the protocols were approved by the Ethics Review Board (ERB) and Internal Review Board of the Affiliated Hospital of Nanjing University of TCM (Nanjing, China). The written-informed consent was obtained from each participant. All investigations were conducted according to the principles expressed in the Declaration of Helsinki as well as national/international regulations.

### Patients' information

For the 6 patients, three were male with 5 hips, and the other three patients were female with 3 hips. The mean age of the patients was 36 year-old (range 26-44 years). One patient was with left hip, 3 patients were with right hips, and two patients were on both sides. Among them, three patients (total 3 hips) were steroid-induced femoral head necrosis; Two patients of 4 hips were alcohol-induced femoral head necrosis. One patient with 1 hip was trauma-induced femoral head necrosis. The course of disease ranged from 3 to 15 months, and the mean course time was 6.8 months. Three cases with combined chronic glomerulonephritis were already cured. All the patients were exclude from other internal medical diseases, i.e. hypertension, diabetes, etc.

According to the Association Research Circulation Osseous (ARCO), staging criteria was divided into IIIA of 3 patients and IIIB of 3 patients. Case inclusive criteria: (1) ages from 18 to 60; (2) ARCO stage IIIA-IIIB; (3) a strong will to operate hip-preserving surgery, and generally in fine condition (4) All patients signed informed-consent form with complete follow-up data. Exclusion criteria of cases: (1) The preoperative femoral head collapse over 4 mm; (2) It had preoperative hip osteoarthritis before surgery; (3) Patients who suffered from acute myocardial infarction, cerebrovascular accident, severe trauma or other major surgeries over previous 6 months; (4) The primary diseases extended liver, kidney and hematopoietic system. All of the participants completed follow-up study (Period of follow-up were from 24 to 48 months, with the average period of 36.6 months).

### Operation surgery procedures

**Figure 1:**
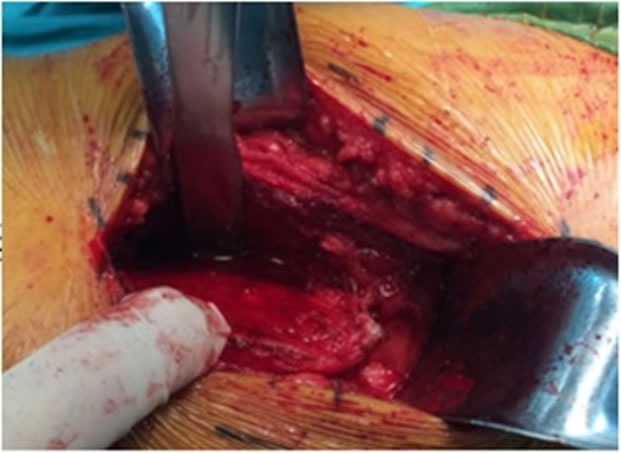
The osteotomy was pulled forward combining with vastus lateralis muscles attached and completely protect rotary muscle

**Figure 2:**
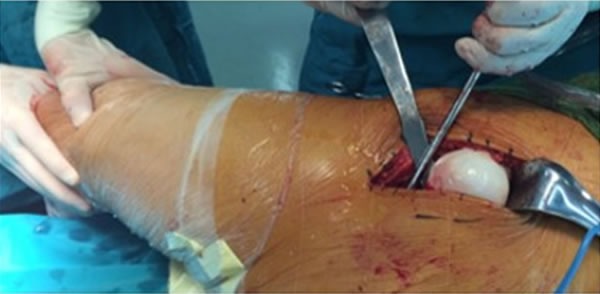
Cut joint capsule by the shape of “Z” to make the femoral head dislocate forward

All patients were operated with general anesthesia in lateral position. Tip of the trochanter shall be set as a center, and the longitudinal incision was about 8 cm outside of the pelvis. We cut tensor fascia lata, separated vastus lateralis muscle from both sides of distal femur, along with upper posterior margin of trochanter and upper margin of shaft of femur. We observed the osteotomy of trochanter along this line from backward to forward (thickness of osteotomy was not exceed 1.5 cm). The osteotomy was pulled forward together with attached vastus lateralis muscles to completely protect rotary muscle (Figure 1). Then we dissected bluntly along the vertex of greater trochanter to anterior superior. The joint capsule can be cut by the shape of “Z”, and flexed mildly, therefore turning outward to make the femoral head dislocate forward. (Figure 2).

**Figure 3:**
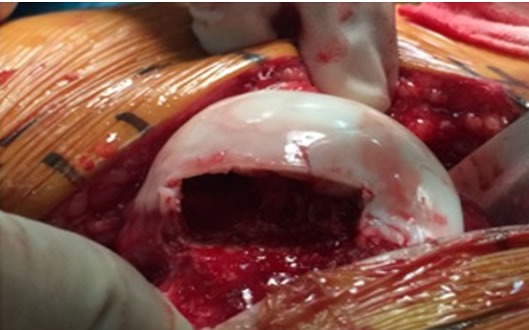
Windowing between the head and neck part under the front lateral necrosis area

After observing the femoral head, the cartilage of necrosis area was found to be located in the front lateral weight-bearing area with obvious deformation and collapse, windowing between the head and neck part under the front lateral necrosis area. The sequestrum would be eliminated completely till the cartilage under the direct view. The autogenous spongy bone were withdrawn for the impacting bone graft at the osteotomy of trochanter major, according to the range of the necrosis (Figure 3). After the completion of the bone graft, the cartilage in the original collapsed area had basically recovered to the normal state, and the blood supply of the femoral head is favorable. The original position of cortical bone of head parts should be coated, and 4.0 mm hollow rivets can be fixed there. After reduction of hip joint, there was no obvious sense of impact after the exercise in all directions. Reduction of the trochanteric osteotomy and the operative incision were washed and sutured, after 4.00 mm hollow rivets fixed in the direction of lesser trochanter.

### Observation index and therapy criteria

Third and sixth months after surgery, all the patients were subjected to X-ray (anteroposterior position and frog position of hip) examination and CT scan. The last follow-up was to examine whether there was collapse of the femoral head, to examine healing process of osteotomy and to evaluate the Harris hip scores. The Harris hip scores in the sixth month after the operation: excellent meant over 90; good meant 70-89 and poor meant less than 70.

### Statistical approach

Mean ± standard deviation (SD) was adopted to describe the continuous variables, and the matched T examinationwas applied to reflect the differences among the groups. The normality test was taken for the difference values before and after the operation, and the results were normalized to the normality. The inspection level α = 0.05. The SAS v9.1.3 (SAS Institute, Cary, North Carolina) statistical software was applied.

## RESULTS

The surgical operations for all patients went completed smoothly without abnormal conditions or serious complications. The operation time was between 86 to 128 minutes, with an average time of 98.8 minutes. The hemorrhage during operation was 320 to 970 mL, with an average of 568.8 mL. All the patients were given skin traction for six weeks in the operation side, as well as 30-minute treatment of vein pump for lower limbs. We also guided the patients to take long-term exercises in quadriceps femoris and musculus gastrocnemius, along with other regular medical care.

Three months after the operation, the patients were burdened on the lower parts with walking aid. Six months after the operation, they were burdened by themselves. One patients with 1 hip suffered from fever and pain around the surgical site two months after surgery. The patient was totally relieved after given anti-inflammation and painkilling treatments. There was no other postoperative complications, i.e. deep venous thrombosis, deep infection, non-healing of the osteotomy and others. The length of stay in hospital was from 14 to 31 days, with an average of 19.6 days.

**Figure 4:**
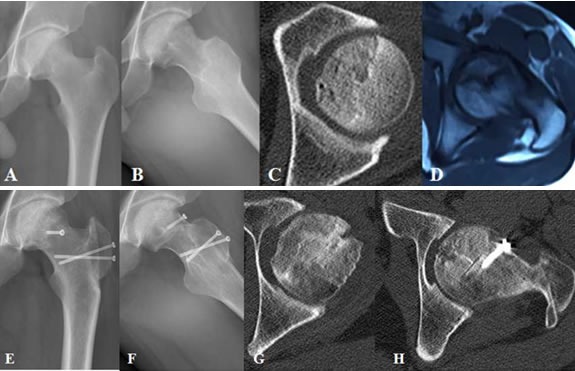
Thirty two-year male patient with alcohol-induced femoral head necrosis of left hip **A.** postoperative view (PA view) of left hip pre-operation; **B.** Frog position of left hip pre-operation; **C.** Necrosis area and collapse of femoral head (FH) shown in CT scan of left hip pre-operation; **D.** Crescent sign shown in MRI of left hip pre-operation; **E.** P-A view of left hip 6-months post-operation; **F.** Frog position of left hip 6-months post-operation; **G.** Bony healing of bone graft and no collapse of FH shown in CT scan 6-months post-operation; H. Bony healing of osteotomy in greater trochanter shown in MRI 6-months post-operation.

All the six patients were followed up. The follow-up time is from 24 to 48 months, with an average of 36.6 months. Until the last follow-up visits, the X-ray and CT film scan showed that all the osteotomy were bony healing (Figure 4). All the patients can be burdened on the ground. One patient had collapse more than 4 mm in 1 hip of femoral head. We then stopped the patient's motion, and anti-inflammation and painkilling treatment were given to the patient. The patient's symptoms were then relieved. This patient was preparing for prosthetic replacement operation.

The preoperative Harris hip scores was 42-68 points with an average of 54.52 ± 8.16 points. The Harris hip score in the last follow-up was around 56-94 points with an average of 80.53 ± 7.62 points, which added 26.01 ± 5.10 points in average. This difference had statistical significance (P <0.001). The curative effects evaluated at the last follow-up were: four patients with 5 hips were excellent; One patient with 2 hips was good; One patient with 1 hip was bad.

## DISCUSSION

The blood vessel distribution of femoral head and femoral neck are often complicated. The blood supply of the burdened area of femoral head is from inside artery of femoral circumflex through autopsy [9, 10]. The blood supply of femoral head can also be fully supplied by upper tenaculum arteries [9, 10]. The inside epiphysis artery only governs the area around fossa ovails rather than other important areas of femoral head [9, 10]. Metaphsis artery and outside artery of femoral circumflex have little or no contribution to blood supply of femoral head. For the hip joint surgical operations, the most important part of inside artery of femoral circumflex is the periphery part of joint capsule of deep branch. Protection of the deep branch of inside artery of femoral circumflex can avoid ANFH, which also becomes the theoretical basis of surgical dislocation of the hip.

The surgical dislocation of hip has been practiced first by Ganz et al., in 2001 [8], which made the acetabular labrum, acetabulum cartilage surface and femoral head visible. At present, the surgical dislocation of hip has been widely utilized in treating femoroacetabular impingement and acetabulum fracture, among others.

The treatment for ANFH is yet varying pending on its progressiveness of the disease [11]. Treatment options include conservative treatment, femoral head heart center decompression impacting bone graft [12, 13], autogenous bone transplant with blood vessels [14, 15], blood vessel bundle implanting [16] as well as rotating osteotomy [17–20]. But the above operations do not have definite therapeutic effect for patients with middle- and advanced-stage femoral head necrosis. Lieberman et al. reviewed 54 literatures of hip-protection surgery for femoral head necrosis from 1998 to 2010, the failure rate of other hip protection methods for collapsed-femoral head necrosis patients has reached 39.8%-100% [21].

For grade IIIA-IIIB ANFH patients, the following are advantages when using our procedure: First, it can effectively protect the blood supply for femoral head. The fast majority of blood supply of the femoral head comes from the ascending limb of extracapsular arterial ring [9, 10]. When there is hip joint dislocation, the blood supply is protected by the complete obturator externus [9, 10]. Therefore, even there is surgical dislocation of hip joint, the blood supply of the femoral head is obviously not influenced. Second, it provides clear vision for sequestrum clearance and impacting bone graft. The current procedure can safely dislocate hip joint, this would let the operators observe collapse degrees and cartilage break conditions of femoral head. This could also let the sequestrum to be cleared thoroughly in the necrosis area, as well as sufficiently impact bone graft and thus provide supporting physically.

Third, this current procedure shall provide sufficient amount of autologous cancellous bone. The conventional hip-protection operation requires autologous bone from ilium or allogeneic bone. Our method used the large amount of autologous cancellous bone in greater trochanter osteotomy area. Thus, sufficient bone should be obtained. It shall also obtain bone healing of bone graft to the maximum degrees in the necrosis area. Fourth, the blood supply in the greater trochanter area is rich. There would be no special concern for the bone healing problems in greater trochanter osteotomy and head neck window place. Fifth, for the femoral head necrosis patients combined with acetabulum impact syndrome, the surgical dislocation of hip can treat the femoral head necrosis and acetabulum impact syndrome simultaneously.

Certainly, the surgical dislocation of the hip for femoral head necrosis is only a treatment option for the already collapsed patients. Because the head neck is windowed and the bone graft is closed to subchondral bone, there always be the situations where the above procedure can't be operated. There are also possibilities that the bone graft may be absorbed or even continual collapsed. This method also has the risk of osteotomy displacement, deformity union and even non-healing.

There are strong theoretical supports for our surgical procedure for high-grade ANFH patients, there is little or no clinic report for this method yet. The mid- to long-term curative benefits of this method awaits to be observed. For the young IIIA-IIIB ANFH patients, our results suggest that they might warrant an opportunity to the current procedure as a safe and effective attempt to delay the disease progression.
